# Long-term prognostic impact of chromosome 1 abnormalities in newly diagnosed multiple myeloma patients: a monocentric study

**DOI:** 10.1007/s00277-026-07072-3

**Published:** 2026-05-19

**Authors:** Nicolas Thomas Iannozzi, Gabriella Sammarelli, Andrea Poletti, Giannalisa Todaro, Matia Bernardi, Matteo Scita, Anna Benedetta Dalla Palma, Vincenzo Raimondi, Oxana Lungu, Eleonora Carboni, Stefania Ricci, Camilla Sitzia, Mattia Dessena, Rosanna Vescovini, Denise Toscani, Paola Storti, Nicola Giuliani

**Affiliations:** 1 Laboratory of Hematology, Department of Medicine and Surgery, University of Parma, Parma, Italy; 2 Hematology and BMT Unit, “Azienda Ospedaliero-Universitaria di Parma”, Parma, Italy; 3 Department of Onco-Hematology-Multiple Myeloma and Monoclonal Gammopathies Program, “Azienda Ospedaliero-Universitaria di Parma”, Parma, Italy

**Keywords:** Cytogenetic abnormalities, Multiple myeloma, Prognosis, Survival

## Abstract

The presence of chromosome 1 abnormalities in multiple myeloma (MM) has been included in the more recent prognostic staging system; however, the prognostic role of chromosome 1 abnormalities remains controversial. Thus, in this study we have investigated the prognostic role of chromosome 1 abnormalities in a cohort of newly diagnosed MM (NDMM), performing a retrospective monocentric analysis of 95 patients. Median follow up was 66 months for overall survival (OS) and progression free survival (PFS), and 55 months for time to next treatment (TTNT). Univariate analyses showed that 1q gain or amplification did not significantly affect outcomes (OS *p*=0.30; PFS *p*=0.69; TTNT *p*=0.91). Conversely, del(1p) was associated with inferior OS (*p*=0.015), PFS (*p*=0.007), and TTNT (*p*=0.038), although this effect did not persist in multivariable models. Notably, the co occurrence of del(1p) and del(17p) identified a subgroup with markedly worse prognosis across all endpoints and represented the only combination with a consistent additive adverse effect. Multivariable analyses confirmed that patients harboring both abnormalities had significantly poorer outcomes, including reduced OS (HR 6.97, 95% CI 2.94–16.54), PFS (HR 6.92, 95% confidence interval [CI] 2.89–16.55), and TTNT (HR 5.21, 95% CI 1.69–16.05. In conclusion, our study suggests that among the cytogenetic abnormalities, del(1p) is a significant negative prognostic factor in NDMM patients in combination with the presence of del(17p), in shaping the prognosis of NDMM.

**Supplementary Information:**

The online version contains supplementary material available at 10.1007/s00277-026-07072-3.

Multiple myeloma (MM) is plasma cell (PC) malignancy characterized by the accumulation of MM cells within the bone marrow [1]. Despite major therapeutic advances over the past decades, a substantial proportion of patients, harboring high-risk genomic features, continues to experience early relapse and inferior survival. Accurate identification of high-risk and the implementation of risk-adapted therapeutic strategies are therefore essential to optimize survival outcomes.

Prognostic assessment in MM patients is guided by the revised international staging system ISS (R-ISS), which includes three chromosomal abnormalities, t(4;14), t(14;16), and del(17p), associated with adverse prognosis, along with ISS stage and LDH level in newly diagnosed MM (NDMM). More recently, the European Myeloma Network (EMN) proposed a second revision of the current R-ISS (R2-ISS), to expand the definition of high-risk disease, including, in addition to IgH translocations and del(17p), the chromosome 1 abnormalities, gain/amp(1q+) and del(1p), recognized to confer poor outcomes, highlighting the particularly unfavorable prognosis associated with their co-occurrence [2]. The possible prognostic role of chromosome 1 aberration has recently been also confirmed by the International Myeloma Working Group (IMWG) which formulated a consensus genomic staging of high-risk MM. According to this framework, patients are considered high-risk if they present: del(17p) with a clonal fraction > 20% and/or TP53 mutation; an IgH translocation [t(4;14), t(14;16), or t(14;20)] in combination with 1q+ and/or del(1p); monoallelic del(1p) together with 1q+ or biallelic del(1p); or β2-microglobulin ≥ 5.5 mg/L with normal creatinine (< 1.2 mg/dL) [3].

Nevertheless, results on the prognostic role of chromosome 1 abnormalities are still discordant [4–9].

For this reason, in this study we investigated the prognostic role of chromosome 1 abnormalities in NDMM, performing a retrospective monocentric analysis of 95 NDMM patients between 2017 and 2022. All patients had available baseline fluorescence in situ hybridization (FISH) data and clinical follow-up. Treatments included proteasome inhibitors, immunomodulatory drugs, and autologous stem cell transplantation (ASCT) in transplant-eligible patients according to standard clinical practice, outside clinical trials (Supplementary Table S1).

Interphase FISH was performed on purified PCs at diagnosis to assess high-risk cytogenetic abnormalities, including del(17p) (TP53), del(1p) (CDKN2C), gain(1q) and amplification of 1q (CKS1B), t(4;14), and del(13q) (RB1). A uniform cutoff of 20% of cells was applied. Gain(1q) was defined as three copies and amplification of 1q as four or more copies.

To evaluate the combined effect of chromosome 1 alterations with other high-risk lesions, paired cytogenetic variables were constructed as four category variables: “both alterations”, “only alteration A”, “only alteration B”, or “none”. Only pairs involving at least one chromosome 1 alteration were analyzed. Variables with fewer than five patients in the “both” categories were excluded. Overall survival (OS), progression-free survival (PFS), and time to next treatment (TTNT) were analyzed using Kaplan-Meier estimates and log-rank tests, with pairwise comparisons when appropriate.

The median follow-up of the study was 66 months for OS and PFS, and 55 months for TTNT. Cox proportional hazards models were used for univariate and multivariable analyses, adjusting for ASCT, ISS and best response. All tests were two-sided, and *p*-values < 0.05 were considered statistically significant.

Baseline clinical and cytogenetic characteristics of the cohort are summarized in Supplementary Table S1. High-risk cytogenetic abnormalities, as reported in Supplementary Table S1, were defined by the presence of at least one of the following lesions: del(17p) with a > 20% cutoff, t(4;14), t(14;16), or t(14;20) [10].The prevalence of cytogenetic abnormalities was as follows: t(4;14) in 8.4%, t(14;16) in 2.1%, t(14;20) in 1.1%, del(13q) in 47.4%, del(17p) in 18.9% (cut-off > 20%), del(1p) in 21.1%, gain(1q) in 45.3%, and amp(1q) in 16.8% (Supplementary Table S1). To better characterize the genomic landscape of the cohort, we also evaluated the co-occurrence patterns among the main cytogenetic abnormalities (Supplementary Figure S1). Del(17p) frequently co-occurred with del(1p) (*n* = 7) and gain(1q) (*n* = 10), while del(1p) showed substantial overlap with gain(1q) (*n* = 16) and del(13q) (*n* = 14). Gain(1q) was the most common abnormality and co-segregated with del(13q) in 21 cases and with amp(1q) in 16 cases (Supplementary Figure S1). In univariate analyses, the presence of the gain/amp(1q) alteration did not demonstrate a significant negative impact when present as a single-variable stratification (OS *p* = 0.30; PFS *p* = 0.69; TTNT *p* = 0.91; Figure 1a-c).

**Fig. 1:**
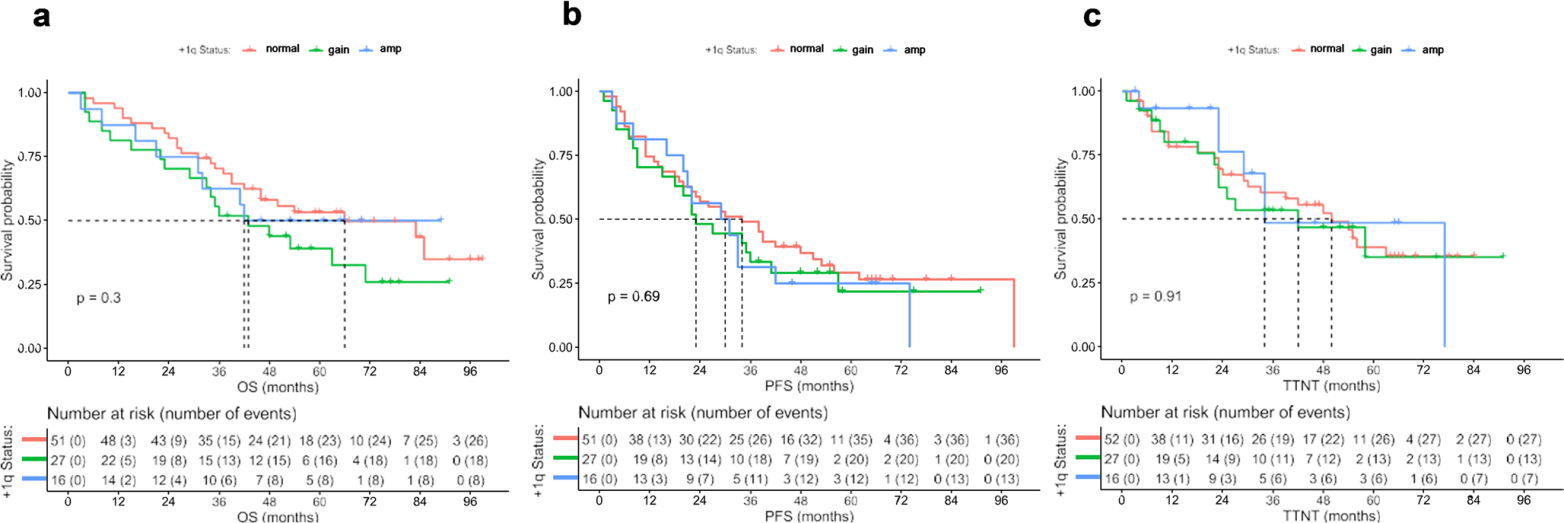
Impact of chromosome 1q status on clinical outcomes. Kaplan-Meier survival curves stratified by chromosome 1q status, classified as normal (red), gain (green), or amplification (blue). Panel **a** shows OS, panel **b** shows PFS, and panel **c **shows TTNT. Dashed horizontal and vertical lines indicate median survival times when reached. *P*-values are derived from log rank tests comparing survival distributions across groups. Numbers at risk and number of events at each time point are reported below each plot. OS=overall survival; PFS=progression free survival; TTNT=time to next treatment; amp=amplification

Conversely, in univariate analyses, del(1p) as a single-variable stratification was associated with significantly shorter OS (*p* = 0.015), PFS (*p* = 0.007), and TTNT (*p* = 0.038) (Fig. 2a-c). A sub-analysis evaluating the combined effect of del(1p) and del(17p) confirmed a pronounced negative impact on PFS and OS (both *p* < 0.0001; Fig. 2d-e), as well as on TTNT (*p* = 0.015; Fig. 2f).

**Fig. 2:**
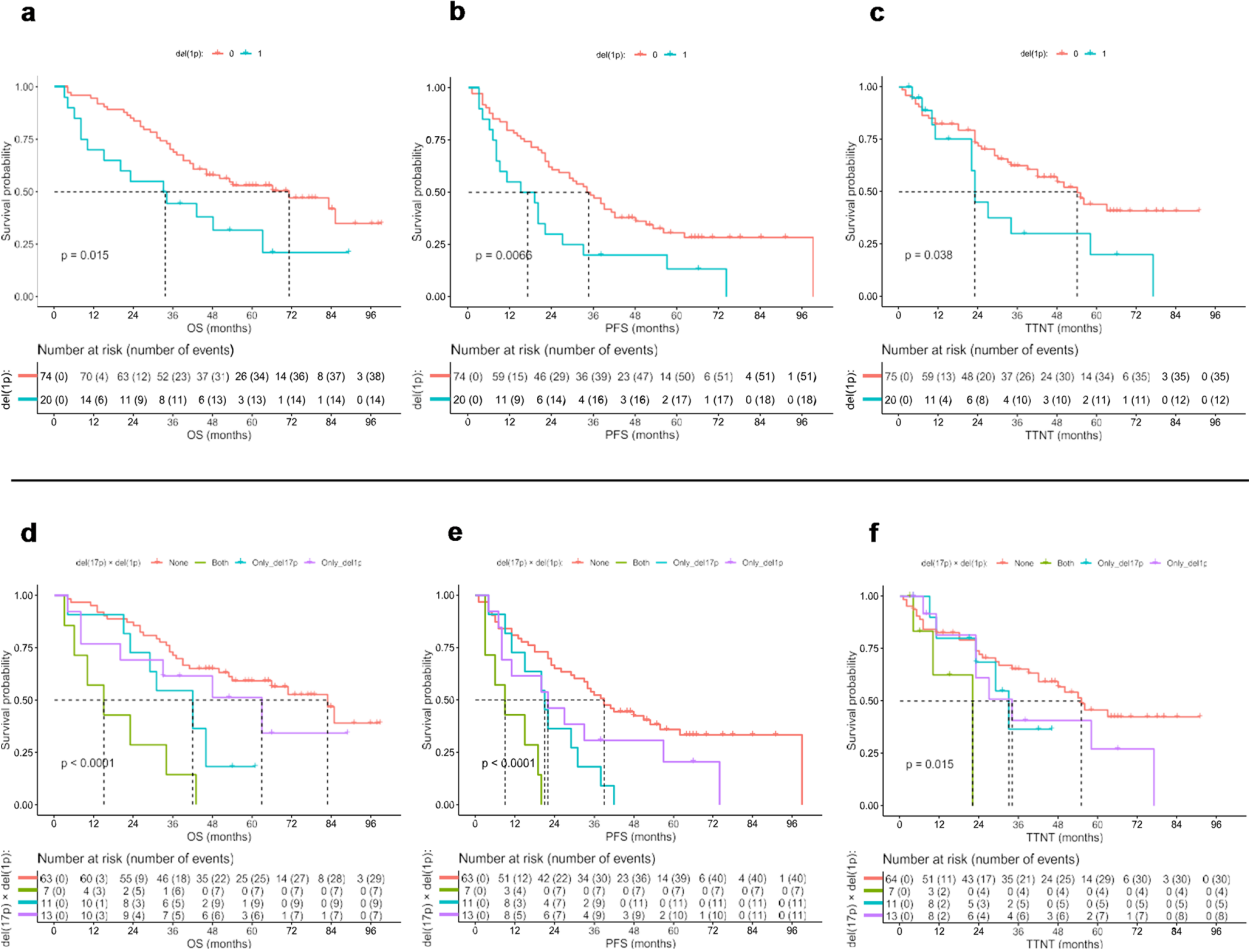
Impact of del(1p) and combined del(17p) and del(1p) on clinical outcomes. Kaplan-Meier survival curves stratified by del(1p) status and by combined del(17p) and del(1p). Panels **a** to **c** show OS, PFS, and TTNT according to del(1p) status, classified as absent or present. Panels **d** to **f** show OS, PFS, and TTNT stratified by combined cytogenetic status, defined as none, del(17p) only, del(1p) only, or both del(17p) and del(1p). Dashed horizontal and vertical lines indicate median survival when reached. *P* values were calculated using the log rank test. Numbers at risk and number of events at each time point are shown below each plot. OS=overall survival; PFS=progression free survival; TTNT=time to next treatment; del=deletion

To further explore the prognostic interactions among cytogenetic lesions, we performed a systematic univariate evaluation of all pairwise combinations of alterations involving chromosome 1. We defined an additive effect based on a consistent significant association of the combined group across multiple endpoints (OS, PFS, and TTNT), together with higher hazard ratios (HR) for the combined group compared to the corresponding single-lesion groups. Based on these criteria, most paired cytogenetic combinations did not show a consistent additive prognostic effect. In our several models, the adverse risk was primarily driven by a single-lesion, most frequently del(1p) or del(17p), rather than by their co- occurrence (Supplementary Table S2).

Interestingly, an additive effect was observed only for the combined presence of del(17p) and del(1p): patients harboring both alterations showed markedly inferior outcomes compared to those with single-lesions or no alterations, with significantly increased hazards for OS (HR 6.97, 95% confidence interval [CI] 2.94–16.54), PFS (HR 6.92, 95% confidence interval [CI] 2.89–16.55), and TTNT (HR 5.21, 95% confidence interval [CI] 1.69–16.05).

On the other hand, combinations involving gain(1q) or amp(1q) did not demonstrate a consistent synergistic effect. Although some paired variables reached statistical significance for individual endpoints, the HR for the double hit groups was comparable to or lower than those observed for single alterations and lacked consistency across OS, PFS, and TTNT. These findings indicate that, within chromosome 1 abnormalities, a true double hit phenotype is only observed in the context of concurrent del(17p) and del(1p), whereas other combinations reflect predominantly single-lesion driven risk. To further assess the independent prognostic contribution of del(17p) and del(1p) and other clinical-biological variables, we performed multivariate Cox proportional-hazards models for OS, PFS, and TTNT (Fig. 3a-c).

**Fig. 3:**
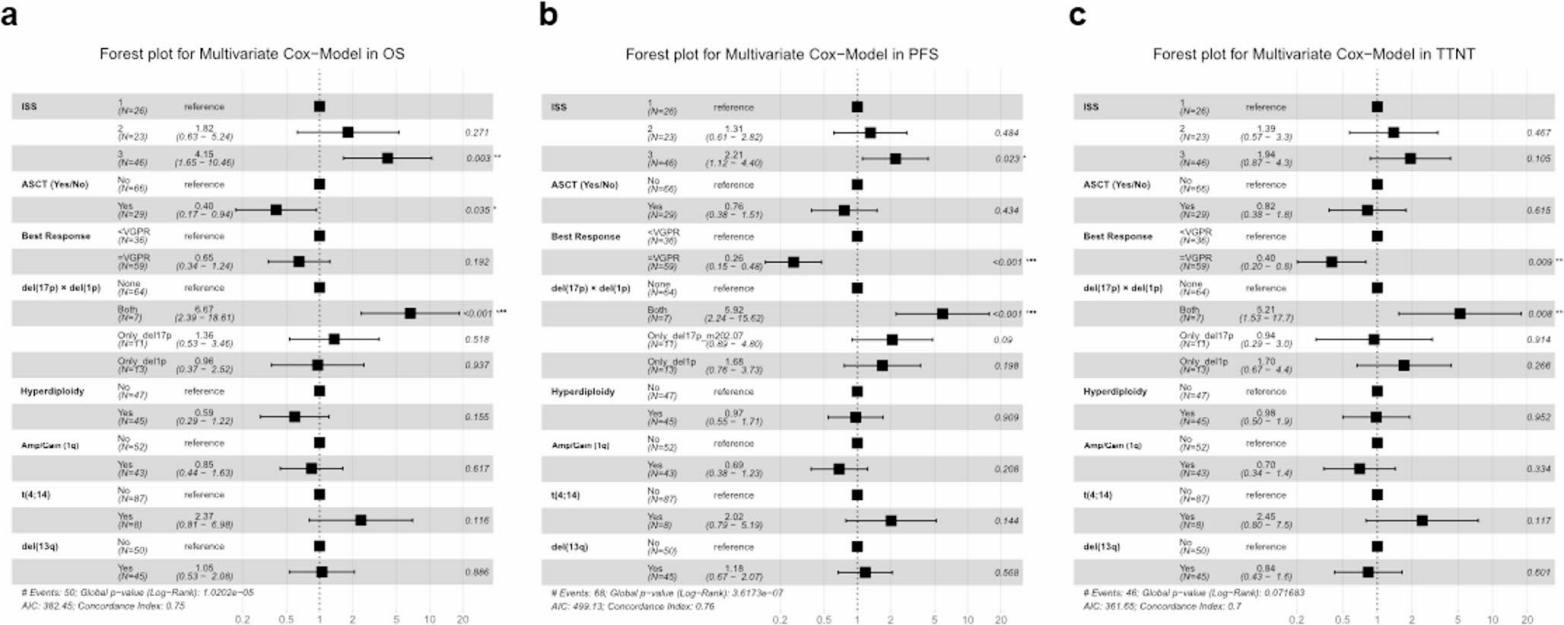
Multivariable Cox regression analysis of clinical outcomes. Forest plots from multivariable Cox proportional hazards models for OS (**a**), PFS (**b**), and TTNT (**c**). Hazard ratios with 95% confidence intervals are shown for each covariate. The reference category for each variable is indicated in the plot. *P* values are derived from Wald tests. Global log rank test *P* values and the number of events included in each model are reported at the bottom of each panel. OS=overall survival; PFS=progression free survival; TTNT=time to next treatment; HR=hazard ratio; CI=confidence interval; ISS=International Staging System; ASCT=autologous stem cell transplantation; VGPR=very good partial response

Across all endpoints, ISS-III and the co-occurrence of del(17p) and del(1p) emerged as the strongest adverse prognostic factors. In the OS model, ISS-III was associated with an increased risk of death (HR 4.15, 95%CI 1.64–10.46), while patients harboring both del(17p) and del(1p) exhibited markedly inferior survival (HR 6.67, 95%CI 2.39–18.61). Undergoing ASCT was independently associated with improved OS (HR 0.40, 95%CI 0.17–0.94).

For PFS, ISS-III and the combined presence of del(17p) and del(1p) remained significantly associated with shorter time to progression (HR 5.92, 95%CI 2.24–15.62), whereas achieving ≥VGPR conferred a substantial protective effect (HR 0.26, 95%CI 0.15–0.48). Similar findings were observed for TTNT, where del(17p) plus del(1p) retained a strong negative impact (HR 5.21, 95%CI 1.53–17.7), and ≥VGPR was associated with prolonged treatment-free intervals.

Interestingly, del(17p) showed a significant impact on OS and PFS but not on TTNT (Supplementary Table S2). This discrepancy may reflect the smaller number of TTNT events, resulting in reduced statistical power, as well as the heterogeneous influence of treatment-related factors on TTNT, which can attenuate the effect of baseline cytogenetics. Notably, the prognostic impact becomes significant when del(17p) co-occurs with del(1p), supporting a cooperative biological effect. Differences between PFS or OS and TTNT may also arise from clinical practice, as treatment initiation is often delayed in biochemical relapse according to IMWG/EMN guidelines, leading to variable alignment between the endpoints.

Other cytogenetic abnormalities, including gain/amp(1q), t(4;14), del(13q), and hyperdiploidy, did not retain statistical significance in the multivariate setting. Collectively, these analyses highlight the synergistic adverse impact of concomitant del(17p) and del(1p), underscoring the biological aggressiveness of this genomic combination.

Our findings clarify the prognostic role of chromosome 1 abnormalities in NDMM. Del(1p) was associated with inferior outcomes in univariate analyses; however, this effect did not persist in multivariable models, indicating that del(1p) alone does not act as an independent prognostic factor in this cohort. Instead, the adverse impact of chromosome 1p loss becomes clinically relevant primarily when it co-occurs with del(17p), a combination that consistently retained significance across all endpoints and emerged as the strongest cytogenetic predictors of poor prognosis [11].

Although the prognostic role of del(1p) has been variably reported in the literature, our findings support its relevance as a biologically meaningful lesion, particularly in the context of its co-occurrence with del(17p), as reflected in updated risk-classification frameworks [3].

A key contribution of our study is the systematic evaluation of co-occurring cytogenetic lesions. The co-occurrence analysis revealed that chromosome 1 abnormalities frequently cluster with other high-risk features, underscoring the genomic complexity of this region. In particular, the combination of del(17p) and del(1p) demonstrated the most pronounced adverse impact across all survival endpoints, both in univariate and multivariate analyses, supporting the concept that patients harboring multiple high-risk lesions represent a biologically aggressive subgroup requiring tailored therapeutic strategies.

Interestingly, gain(1q) and amp(1q) did not demonstrate a significant prognostic effect when present as a single-variable stratification. This finding is in line with some, but not all, published studies and raises the possibility that the adverse impact of 1q+ may depend on co-segregating lesions rather than on copy-number alteration alone [12–15]. The substantial overlap between gain(1q) and other abnormalities in our cohort further supports this interpretation.

Our multivariate analysis confirmed that ISS-III and the combined presence of del(17p) and del(1p) are the dominant prognostic determinants in this cohort. The strong effect of this combination reinforces its inclusion among the high-risk categories in recent classification systems. Conversely, other cytogenetic abnormalities, including t(4;14), del(13q), and hyperdiploidy, did not retain significance, reflecting the evolving landscape of prognostic markers in the era of modern therapy.

This study has several limitations, including its retrospective design, the relatively small sample size, which may affect interaction analyses, and the heterogeneity of treatment regimens. Some cytogenetic combinations were infrequent, thereby limiting the statistical power to detect modest effects. Nevertheless, the consistency of our findings across multiple endpoints and analytical approaches strengthens their validity.

In conclusion, our study suggests that among the cytogenetic abnormalities, del(1p) is a significant negative prognostic factor in NDMM patients in combination with the presence of del(17p), in shaping the prognosis of NDMM.

## Electronic Supplementary Material

Below is the link to the electronic supplementary material.

Supplementary Material 1

## Data Availability

No datasets were generated or analysed during the current study.
