# Supplementary material for: Long-term prognostic impact of chromosome 1 abnormalities in newly diagnosed multiple myeloma patients: a monocentric study
Source: Ann Hematol. 2026 May 19;105(8):325. doi: 10.1007/s00277-026-07072-3 (PMC13350107; doi:10.1007/s00277-026-07072-3)
Supplement: Supplementary file 1 — Supplementary Material 1 [file 277_2026_7072_MOESM1_ESM.docx]

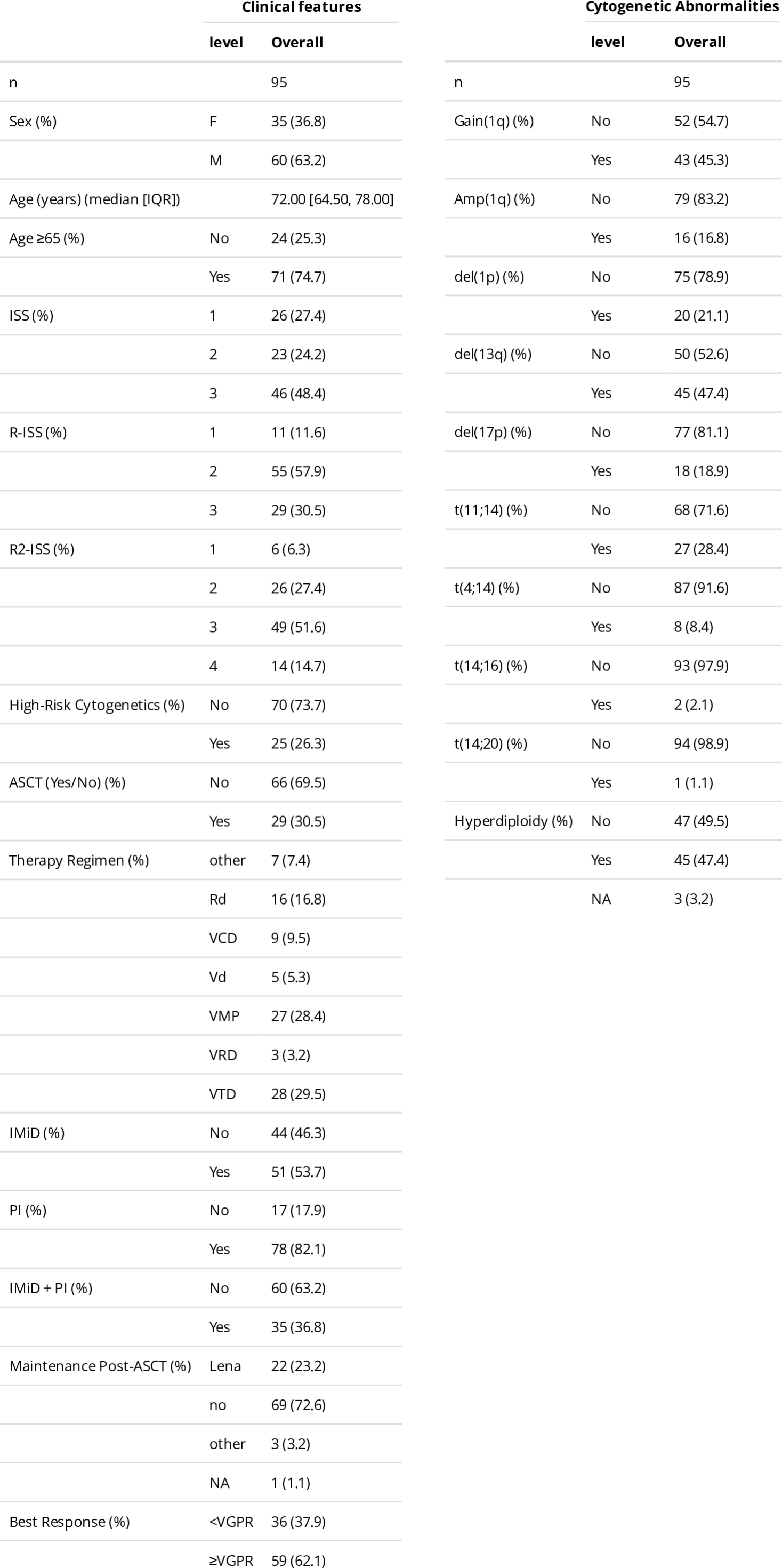


**Supplementary Table S1: Baseline clinical and cytogenetic characteristics of the study cohort.** Clinical features and cytogenetic abnormalities of the 95 patients included in the study are reported. Categorical variables are shown as number and percentage. Continuous variables are reported as median with interquartile range. IQR = interquartile range; ISS = International Staging System; R ISS = Revised International Staging System; R2 ISS = Second Revision of the International Staging System; ASCT = autologous stem cell transplantation; IMiD = immunomodulatory drug; PI = proteasome inhibitor; VGPR = very good partial response; NA = not available; VCD = bortezomib cyclophosphamide dexamethasone; Vd = bortezomib dexamethasone; VMP = bortezomib melphalan prednisone; VRD = bortezomib lenalidomide dexamethasone; VTD = bortezomib thalidomide dexamethasone.


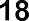

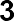

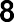

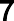

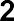

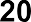

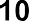

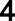

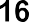

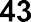

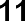

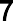

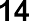

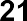

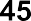

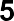

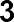

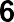

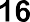

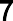

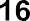

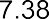

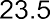

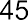


**Supplementary Figure S1: Pairwise co occurrence of cytogenetic abnormalities.** Heatmap showing the number of patients harboring pairwise combinations of recurrent cytogenetic abnormalities. Each cell reports the absolute count of patients with the corresponding combination, with color intensity reflecting the frequency of co occurrence. Diagonal cells represent the total number of patients carrying each individual abnormality.

| **variable** | **group** | **HR** | **conf.low** | **conf.high** | **p.value** | **p.val.code** | **surv** |
| --- | --- | --- | --- | --- | --- | --- | --- |
| **+1q Status** | gain  amp | 1.60  1.12 | 0.87  0.51 | 2.92  2.48 | 0.129  0.780 | ns  ns | **OS**  **OS** |
| **del(17p)** | Yes | 3.38 | 1.83 | 6.23 | 0.000 | *** | **OS** |
| **del(1p)** | Yes | 2.11 | 1.14 | 3.91 | 0.018 | * | **OS** |
| **t(4;14)** | Yes | 1.42 | 0.56 | 3.58 | 0.460 | ns | **OS** |
| **del(13q)** | Yes | 1.41 | 0.81 | 2.43 | 0.220 | ns | **OS** |
| **Gain(1q) x del(1p)** | Both | 2.10 | 1.02 | 4.31 | 0.044 | * | **OS** |
|  |  | 4.01  1.30 | 1.16  0.68 | 13.78  2.50 | 0.028  0.428 | * ns |  |
|  | Only_del1p  Only_gain1q |  |  |  |  |  | **OS OS** |
| **del(17p) x del(1p)** | Both | 6.97 | 2.94 | 16.54 | 0.000 | *** | **OS** |
|  |  | 2.69  1.55 | 1.25  0.68 | 5.79  3.55 | 0.011  0.297 | * ns |  |
|  | Only_del17p  Only_del1p |  |  |  |  |  | **OS OS** |
| **Gain(1q) x t(4;14)** | Both | 1.14 | 0.27 | 4.84 | 0.863 | ns | **OS** |
|  |  | 1.57  2.73 | 0.88  0.81 | 2.79  9.20 | 0.124  0.104 | ns ns |  |
|  | Only_gain1q  Only_t_4_14 |  |  |  |  |  | **OS OS** |
| **Amp(1q) x del(1p)** | Both | 0.72 | 0.17 | 2.99 | 0.646 | ns | **OS** |
|  |  | 1.53  3.56 | 0.63  1.79 | 3.68  7.07 | 0.346  0.000 | ns  *** |  |
|  | Only_amp1q  Only_del1p |  |  |  |  |  | **OS OS** |
| **Gain(1q) x del(17p)** | Both | 4.39 | 1.94 | 9.97 | 0.000 | *** | **OS** |
|  |  | 3.34  1.34 | 1.38  0.69 | 8.09  2.59 | 0.008  0.382 | ** ns |  |
|  | Only_del17p  Only_gain1q |  |  |  |  |  | **OS OS** |
| **Gain(1q) x del(13q)** | Both | 1.90 | 0.84 | 4.31 | 0.126 | ns | **OS** |
|  |  | 2.10  2.15 | 0.96  0.97 | 4.57  4.75 | 0.062  0.059 | .  . |  |
|  | Only_del13  Only_gain1q |  |  |  |  |  | **OS OS** |
| **del(1p) x del(13q)** | Both | 2.36 | 1.10 | 5.03 | 0.027 | * | **OS** |
|  |  | 1.29  2.32 | 0.68  0.79 | 2.45  6.79 | 0.437  0.124 | ns ns |  |
|  | Only_del13  Only_del1p |  |  |  |  |  | **OS OS** |
| **del(1p) x t(4;14)** | Both | 1.41 | 0.19 | 10.34 | 0.735 | ns | **OS** |
|  |  | 2.32  1.78 | 1.22  0.63 | 4.41  5.04 | 0.010  0.277 | * ns |  |
|  | Only_del1p  Only_t_4_14 |  |  |  |  |  | **OS OS** |

| **variable** | **group** | **HR** | **conf.low** | **conf.high** | **p.value** | **p.val.code** | **surv** |
| --- | --- | --- | --- | --- | --- | --- | --- |
| **+1q Status** | gain  amp | 1.22  1.25 | 0.70  0.66 | 2.10  2.36 | 0.483  0.495 | ns  ns | **PFS**  **PFS** |
| **del(17p)** | Yes | 3.20 | 1.81 | 5.64 | 0.000 | *** | **PFS** |
| **del(1p)** | Yes | 2.09 | 1.22 | 3.60 | 0.008 | ** | **PFS** |
| **t(4;14)** | Yes | 1.67 | 0.76 | 3.68 | 0.200 | ns | **PFS** |
| **del(13q)** | Yes | 1.35 | 0.84 | 2.16 | 0.220 | ns | **PFS** |
| **Gain(1q) x del(1p)** | Both | 1.89 | 1.01 | 3.55 | 0.048 | * | **PFS** |
|  |  |  | 1.80  0.64 | 15.92  1.99 | 0.003  0.685 | ** ns |  |
|  | Only_del1p  Only_gain1q | 5.35  1.12 |  |  |  |  | **PFS PFS** |
| **del(17p) x del(1p)** | Both | 6.92 | 2.89 | 16.55 | 0.000 | *** | **PFS** |
|  |  |  | 1.40  0.92 | 5.56  3.50 | 0.003  0.089 | **  . |  |
|  | Only_del17p  Only_del1p | 2.80  1.79 |  |  |  |  | **PFS PFS** |
| **Gain(1q) x t(4;14)** | Both 2.45 | | 0.85 | 7.04 | 0.098 | . | **PFS** |
|  |  |  | 0.72  0.41 | 1.94  4.38 | 0.518  0.635 | ns ns |  |
|  | Only_gain1q 1.18  ` 1.33 | |  |  |  |  | **PFS PFS** |
| **Amp(1q) x del(1p)** | Both | 1.39 | 0.55 | 3.52 | 0.488 | ns | **PFS** |
|  |  |  | 0.64  1.50 | 2.94  5.37 | 0.410  0.001 | ns  ** |  |
|  | Only_amp1q  Only_del1p | 1.38  2.84 |  |  |  |  | **PFS PFS** |
| **Gain(1q) x del(17p)** | Both | 3.43 | 1.63 | 7.23 | 0.001 | ** | **PFS** |
|  |  |  | 1.54  0.68 | 7.85  2.06 | 0.003  0.543 | ** ns |  |
|  | Only_del17p  Only_gain1q | 3.48  1.19 |  |  |  |  | **PFS PFS** |
| **Gain(1q) x del(13q)** | Both | 1.62 | 0.83 | 3.17 | 0.154 | ns | **PFS** |
|  |  |  | 0.72 | 2.69 | 0.318 | ns |  |
|  | Only_del13 | 1.40 |  |  |  |  | **PFS** |

|  | Only_gain1q | 1.27 | 0.65 | 2.50 | 0.481 | ns | **PFS** |
| --- | --- | --- | --- | --- | --- | --- | --- |
| **del(1p) x del(13q)** | Both | 2.50 | 1.28 | 4.85 | 0.007 | ** | **PFS** |
|  |  | 1.16  1.74 | 0.66  0.67 | 2.01  4.51 | 0.610  0.255 | ns ns |  |
|  | Only_del13  Only_del1p |  |  |  |  |  | **PFS PFS** |
| **del(1p) x t(4;14)** | Both | 3.56 | 0.84 | 15.05 | 0.085 | . | **PFS** |
|  |  | 2.09  1.66 | 1.18  0.66 | 3.71  4.21 | 0.012  0.284 | * ns |  |
|  | Only_del1p  Only_t_4_14 |  |  |  |  |  | **PFS PFS** |

| **variable** | **group** | **HR** | **conf.low** | **conf.high** | **p.value** | **p.val.code** | **surv** |
| --- | --- | --- | --- | --- | --- | --- | --- |
| **+1q Status** | gain  amp | 1.10  0.92 | 0.57  0.40 | 2.15  2.11 | 0.769  0.839 | ns  ns | **TTNT**  **TTNT** |
| **del(17p)** | Yes | 2.01 | 0.94 | 4.29 | 0.070 | . | **TTNT** |
| **del(1p)** | Yes | 2.01 | 1.03 | 3.89 | 0.039 | * | **TTNT** |
| **t(4;14)** | Yes | 1.66 | 0.65 | 4.23 | 0.290 | ns | **TTNT** |
| **del(13q)** | Yes | 1.07 | 0.60 | 1.91 | 0.810 | ns | **TTNT** |
| **Gain(1q) x del(1p)** | Both | 1.63 | 0.75 | 3.51 | 0.216 | ns | **TTNT** |
|  |  | 5.60  0.92 | 1.57  0.45 | 19.94  1.88 | 0.008  0.815 | ** ns |  |
|  | Only_del1p  Only_gain1q |  |  |  |  |  | **TTNT TTNT** |
| **del(17p) x del(1p)** | Both | 5.21 | 1.69 | 16.05 | 0.004 | ** | **TTNT** |
|  |  | 1.55  1.67 | 0.59  0.76 | 4.07  3.64 | 0.375  0.201 | ns ns |  |
|  | Only_del17p  Only_del1p |  |  |  |  |  | **TTNT TTNT** |
| **Gain(1q) x t(4;14)** | Both | 3.21 | 1.09 | 9.47 | 0.035 | * | **TTNT** |
|  |  | 0.85  0.51 | 0.46  0.07 | 1.59  3.79 | 0.621  0.510 | ns ns |  |
|  | Only_gain1q  Only_t_4_14 |  |  |  |  |  | **TTNT TTNT** |
| **Amp(1q) x del(1p)** | Both | 1.08 | 0.33 | 3.55 | 0.898 | ns | **TTNT** |
|  |  | 1.02  2.86 | 0.36  1.33 | 2.90  6.15 | 0.971  0.007 | ns  ** |  |
|  | Only_amp1q  Only_del1p |  |  |  |  |  | **TTNT TTNT** |
| **Gain(1q) x del(17p)** | Both | 2.21 | 0.81 | 5.99 | 0.120 | ns | **TTNT** |
|  |  | 1.74  0.95 | 0.59  0.50 | 5.14  1.83 | 0.316  0.880 | ns ns |  |
|  | Only_del17p  Only_gain1q |  |  |  |  |  | **TTNT TTNT** |
| **Gain(1q) x del(13q)** | Both | 1.18 | 0.56 | 2.48 | 0.664 | ns | **TTNT** |
|  |  | 0.76  0.70 | 0.35  0.30 | 1.66  1.62 | 0.492  0.400 | ns ns |  |
|  | Only_del13  Only_gain1q |  |  |  |  |  | **TTNT TTNT** |
| **del(1p) x del(13q)** | Both | 2.18 | 0.99 | 4.79 | 0.053 | . | **TTNT** |
|  |  | 0.83  1.31 | 0.42  0.39 | 1.65  4.39 | 0.591  0.666 | ns ns |  |
|  | Only_del13  Only_del1p |  |  |  |  |  | **TTNT TTNT** |
| **del(1p) x t(4;14)** | Both | 4.75 | 1.10 | 20.50 | 0.037 | * | **TTNT** |
|  |  | 1.85  1.35 | 0.91  0.41 | 3.79  4.43 | 0.091  0.625 | .  ns |  |
|  | Only_del1p  Only_t_4_14 |  |  |  |  |  | **TTNT TTNT** |

**Supplementary Table S2: Univariable Cox regression analyses for cytogenetic HR variables and interactions variables.** Results of univariable Cox proportional hazards models for OS, PFS, and TTNT. For each variable, hazard ratios with 95 percent confidence intervals, P values, and significance codes are reported. OS = overall survival; PFS = progression free survival; TTNT = time to next treatment; HR = hazard ratio; CI = confidence interval.
